# Variables associated to intensive care unit (ICU)-mortality among patients admitted to surgical intensive care unit in Ethiopia: a retrospective observational study

**DOI:** 10.1186/s12871-023-02230-w

**Published:** 2023-08-18

**Authors:** Misgan Mulatie Zewudie, Debas Yaregal Melesse, Tesera Dereje Filatie, Mulualem Endeshaw Zeleke

**Affiliations:** 1 Department of Anesthesia, College of Medicine and Health Sciences, Injibara University, Injibara, Ethiopia; 2 Department of Anesthesia, College of Medicine and Health Science, University of Gondar, Gondar, Ethiopia

**Keywords:** Incidence, Intensive care unit, Mortality, Predictors, Surgical

## Abstract

**Background:**

The present study aimed to assess variables associated to ICU-mortality among patients admitted to surgical intensive care unit in Ethiopia.

**Methods:**

A Hospital-based retrospective follow-up study was conducted on all patients who were admitted to the surgical intensive care unit. Data were extracted from patients’ charts with a pretested data extraction tool, entered into Epi-data 4.6.0, and analyzed with STATA- 14. Bivariate and multivariate Cox proportional hazards regression models were fitted.

**Results:**

Of the total study participants (388), 148 (38.1%) patients admitted to the surgical intensive care unit died during the follow-up period with a median survival time of 11 days. Potassium level < 3.5 mmol/L (adjusted hazard ratio ( AHR): 3.46, 95% CI (1.83 6.55), potassium level > 5.0 mmol/L (AHR:2.41, 95% CI (1.29–4.51), hypoxia (AHR:1.66, 95% CI (1.10–2.48), Glasgow Coma Scale (GCS) score < 9 (AHR: 4.06, 95% CI (1.51–10.89), mechanical ventilation (AHR:12, 95%CI (3–45), absence of thromboprophylaxis (AHR:10.8,95% CI (6.04–19.29), absence of enteral feeding (AHR:3.56, 95% CI (2.20–5.78) were variables associated with ICU-mortality among patients admitted to surgical intensive care unit.

**Conclusions:**

The overall ICU-mortality of patients admitted to our surgical intensive care unit was higher compared to patients admitted to similar intensive care unit in developed countries. The variables associated to ICU-mortality among patients admitted to surgical intensive care unit were abnormal serum potassium level, lower GCS score, mechanical support, hypoxia, absence of thromboprophylaxis, and enteral feeding.

**Supplementary Information:**

The online version contains supplementary material available at 10.1186/s12871-023-02230-w.

## Background

The intensive care unit (ICU) is where patients that need critical care are managed. The surgical intensive care unit (SICU) is one of the acute care facilities with a focus on surgical conditions [1]. Each year, 310 million major surgical procedures are performed worldwide [2]. Nearly seven million patients experienced serious morbidity during the perioperative phase, yet only 0.5% of surgeries result in death [3, 4]. According to the intensive care over nations (ICON) audit, the likelihood of death is statistically significantly correlated with per-country income [5].

The risk of death in critical care units is determined by a number of measures, including the Simplified Acute Physiology Score (SAPS) and the Acute Physiology and Chronic Health Evaluation (APACHE) score [6].

In affluent nations, the ICU-mortality in surgical intensive care units ranges from 9.3 to 26.2% [7, 8] while in undeveloped nations, varies from 27 to 53.6% [9–12]. Between 35.4 and 46.3% of patients in surgical intensive care units in Ethiopia die, while 36.5 to 47% of patients experience complications, with organ failure being the most frequent [13–15].

Trauma from a road traffic accident (RTA) was the most common cause of admission to a surgical intensive care unit (SICU) in Ethiopia, and surgical cases in general accounted for 22.1% of intensive care unit admissions, with males having a higher admission rate than females [16]. Developing quality indicators, monitoring resources, conducting audits, and making adjustments should all be requirements for providing high-quality care [17].

Patients usually get vasopressor support and mechanical ventilation in the surgical intensive care unit (SICU) [5, 18, 19].

Infection, trauma, and a lack of essential medications and supplies contribute to a greater ICU-mortality among patients referred to surgical intensive care units [14, 19]. Resources (personnel, equipment, drugs), patients’ prior medical histories, and other factors can also have a direct impact on ICU-mortality in critical care units in hospitals around the world [20].

The nation’s first critical care unit was established in Addis Abeba’s Tikur Anbessa Hospital more than 30 years ago, and since then, both public and private hospitals have begun to offer more intense care services. The country’s earlier studies focused on the admission trends, indications, and risk factors for ICU-mortality in the medical critical care unit [21, 22].

This study aimed to investigate variables associated to ICU-mortality among patients admitted to surgical intensive care unit in Ethiopia.

## Methods

### Study design, period, and setting

A single-centered retrospective follow-up study was conducted, from September 19/2019- April 30/2022 G.C, at the University of Gondar Comprehensive Specialized Hospital. University of Gondar Comprehensive Specialized Hospital is providing services for more than 7 million people. On September 19, 2019 (G.C.), the surgical intensive care service was launched with four beds, two mechanical ventilators, one defibrillator, and four non-invasive hemodynamic monitoring gadgets. Ten functional beds, four mechanical ventilators with functional monitoring, a staff of three anesthesiologists, one resident in general surgery who rotates monthly, one general practitioner, and 12 critical care nurses make up the surgical intensive care unit (SICU) at the moment. The area provides critical care services for surgical, trauma and orthopedics, and obstetric cases.

### Study population and data source

**Fig. 1:**
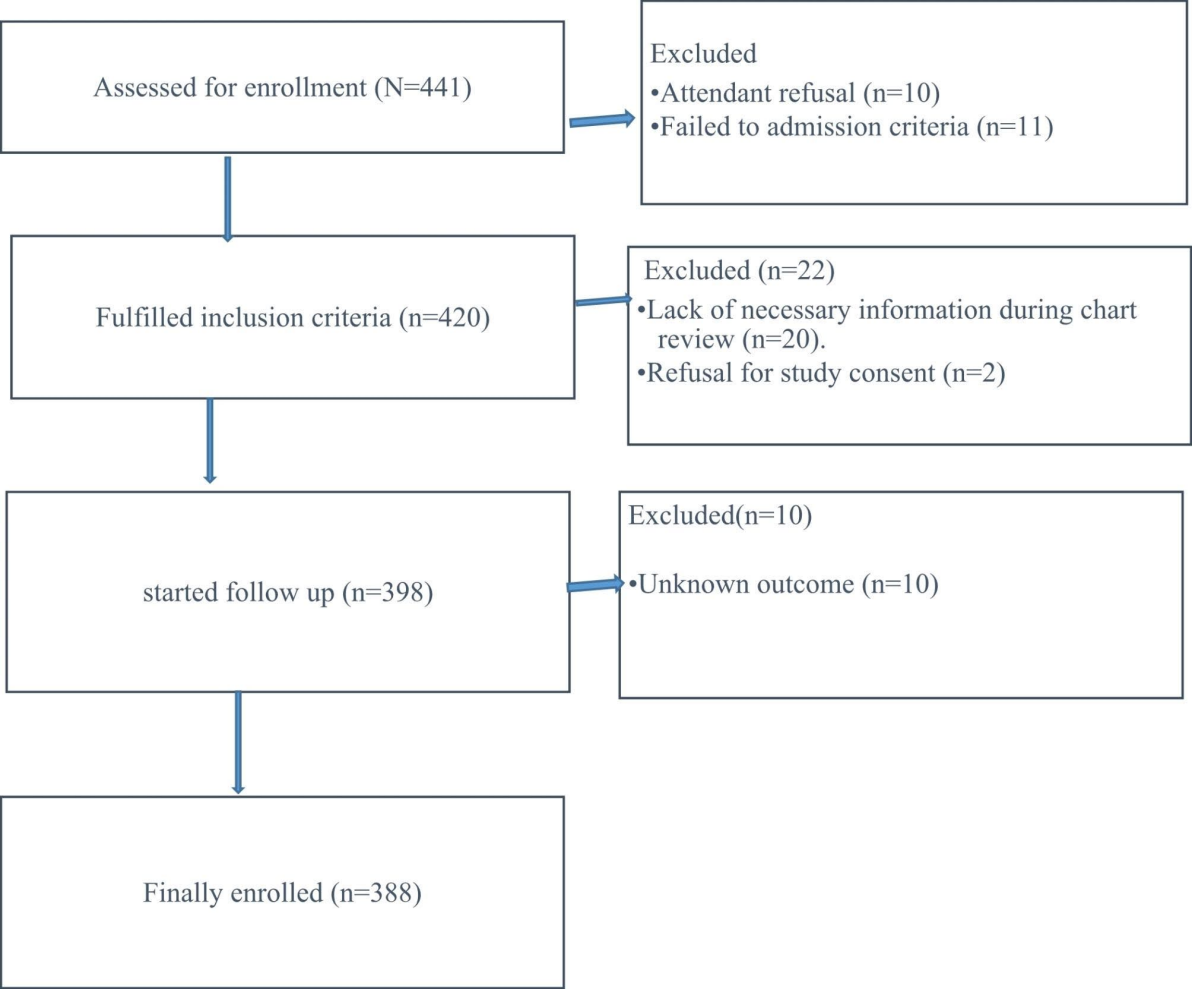
STROBE diagram shows the study participants who were included and excluded in the study (STROBE: Strengthening the reporting of observational studies in epidemiology)

The study included all patients who met the requirements for admission to the SICU, including those who needed or were likely to need advanced respiratory support, needed support for two or more organ systems, or had chronic impairment of one or more organ systems who also needed support for an acute reversible failure of another organ. Patients whose outcomes were unknown were excluded from the study and reported with, ‘‘The Strengthening the Reporting of Observational Studies in Epidemiology (STROBE) statement: guidelines for reporting observational studies’’ [23](Fig. 1). A pretested structured questionnaire that included the chart numbers of patients, date of admission, socio-demographic characteristics, source of admission, diagnosis at admission, admission category, vital signs at admission, presence of comorbidity, length of stay, intervention in surgical intensive care unit, and outcome was used to collect the data. The University of Gondar’s School of Medicine’s institutional review board (IRB) gave its approval to this study with the reference/number/ SoM/12/02/2022. The study was carried out in accordance with the Declaration of Helsinki. Documentation of informed consent was waived by our institutional review board, University of Gondar, School of Medicine.

### Dependent and independent variables

The outcome variable/dependent variable was ICU-mortality in the surgical intensive care unit (number of patients in the surgical intensive care unit of the setting (University of Gondar) who passed away), while the independent variables were age, sex, residency, type of surgery (elective surgery, emergency surgery, and traumatic injury), operated, not-operated, cancer, cardiac illness, hypertension, diabetes mellitus (DM), asthma, chronic obstructive pulmonary disease (COPD), oxygen saturation (SpO2), temperature, glasgow coma scale (GCS) score, creatinine, serum glutamic pyruvic transaminase (SGPT) level, serum glutamic-oxaloacetic transaminase (SGOT), random blood sugar (RBS), hemoglobin (Hgb), mechanical ventilation, vasopressors, blood transfusion, administration of thromboprophylaxis, feeding, and fluid administration, cardiac arrest, anemia, arrhythmia, infection, and aspiration.

### Statistical analyses

Epi-Data version 4.6.0 was used to enter the data, and STATA version 14 was used for the analysis. Categorical variables were expressed as proportions and numerical descriptive statistics were expressed as mean with standard deviation (SD) or median with interquartile range (IQR).

The log-rank test and Kaplan-Meier survival curve were fitted to the ICU-mortality of patients in surgical intensive care unit to evaluate whether there was a difference in the ICU-mortality among the participants. To find the variables associated with the ICU-mortality, bivariate and multivariate survival analyses were performed. Variables with a p-value of 0.25 in the bivariable analyses were candidates for the multivariable analyses. An adjusted hazard ratio (AHR) with a 95% confidence interval was used to assess the strength of the association between the outcome and independent variables. In multivariable analyses, an independent variable was considered statistically significant if its p-value was < 0.05.

## Results

### Demographic characteristics and admission patterns of the study participants

Among the 441 participants, 388 (87.98%) patients admitted in surgical intensive care unit were included in the final analysis. Two hundred eighty of the study participants were males, and the majority of participants, 257 (66.2%), were from rural areas. Nearly half (49.7%) of the participants were aged between 15 and 30 years and had a 49.3% of ICU-mortality (Table 1).

**Table 1:** Socio-demographic characteristics and ICU-mortality of patients in SICU, (N = 388)

Variables	Categories	Total (N = 388)	Outcome	
			Dead (n = 148)	Alive (n = 240)
Sex	Female	108 (27.8%)	32 (21.6%)	76 (31.7%)
	Male	280 (72.2%)	116 (78.4%)	164 (68.3%)
Age (years)	15–30	193 (49.7%)	73 (49.3%)	120 (50%)
	31–45	84 (21.6%)	38 (25.7%)	46 (19.2%)
	46–64	82 (21.1%)	25 (16.9%)	57 (23.7%)
	≥ 65	29 (7.5%)	12 (8.1%)	17 (7.1%)
Residence	Urban	131 (33.8%)	48 (32.4%)	83 (34.6%)
	Rural	257 (66.2%)	100 (67.6%)	157 (65.4%)

Key: - SICU = Surgical Intensive Care Unit

Of the total patients enrolled in the study, patients who came from emergency departments to surgical intensive care unit experienced the highest ICU-mortality, 54% (Table 2).

**Table 2:** Admission characteristics and ICU-mortality of patients in SICU, (N = 388)

Variables	Categories	Total (N = 388)	Outcome	
			Dead (n = 148)	Alive (n = 240)
Source of admission	Emergency department	203 (52.3%)	80 (54%)	123 (51%)
	Inpatient	22 (5.7%)	4 (2.7%)	18 (7.5%)
	Operation room	121 (31.2%)	50 (33.8%)	71 (29.7%)
	PACU	42 (10.8%)	14 (9.5%)	28 (11.8%)
Urgency cases	Emergency cases	336 (86.6%)	140 (94.6%)	196 (81.7%)
	Elective cases	52 (13.4%)	8 (5.4%)	44 (18.3%)
Admission unit	Surgery	151 (38.9%)	56 (37.8%)	95 (39.6%)
	Gynecology/Obstetrics	13 (3.4%)	5 (3.4%)	8 (3.3%)
	Orthopedics and trauma	224 (57.7%)	87 (58.8%)	137 (57.1%)

Key: PACU = Post- Anesthesia Care Unit; SICU = Surgical Intensive Care Unit

The commonest indication for admission to SICU was a head injury, 128 (33%). Septic shock was the leading cause of death for patients in surgical intensive care unit, 63 (42.6%). Among patients admitted to SICU, 38 (9.58%) had different types of comorbidities. The commonest complication was aspiration pneumonia (Table 3).

**Table 3:** Indications for ICU admission, co-morbidities, complications, and ICU-mortality of patients in SICU, (N = 388)

Variables	Categories	Total (N = 388)	Outcome	
			Dead (n = 148 )	Alive (n = 240)
Indication	Peritonitis	24 (6.2%)	5 (3.4%)	19 (7.9%)
	Septic shock	114 (29.4%)	63 (42.6%)	51 (21.2%)
	Head injury	128 (33%)	37 (25%)	91 (37.9%)
	Hemorrhagic shock	22 (5.7%)	7 (4.7%)	15 (6.3%)
	Thoracoabdominal injury	49 (12.6%)	14 (9.5%)	35 (14.6%)
	ARDS	51 (13.1%)	22 (14.9%)	29 (12.1%)
Comorbidity	Yes	38 (9.8%)	12 (8.1%)	26 (10.8%)
	No	350 (90.2%)	136 (91.9%)	214 (89.2%)
Type of comorbidity (N = 38)	Hypertension	8 (21.1%)	4 (2.7%)	4 (1.7%)
	HIV	5 (13.2%)	3 (2%)	2 (0.8%)
	Cancer	21 (55.3%)	3 (2%)	18 (7.5%)
	Others (Diabetic, asthmatic.)	4 (10.5%)	2 (1.4%)	2 (0.8%)
Readmission	Yes	3 (0.8%)	0 (0%)	3 (1.2%)
	No	385 (99.2%)	148 (100%)	237 (98.8%)
Complications	Yes	176 (45.4%)	93 (62.8%)	83 (34.6%)
	No	212 (54.6%)	55 (37.2%)	157 (65.4%)
Complications during ICU stay (N = 176)	Arrhythmia	34 (19.3%)	23 (15.5%)	11 (4.6%)
	Aspiration pneumonia	87 (49.4%)	33 (22.3%)	54 (22.5%)
	Cardiac arrest	12 (6.8%)	10 (6.8%)	2 (0.8%)
	Sepsis	24 (13.6%)	14 (9.5%)	10 (4.2%)
	AKI	19 (10.8%)	16 (10.9%)	3 (1.3%)

Key: ARDS = Acute Respiratory Distress Syndrome; HIV = Human Immune Virus; AKI = Acute Kidney Injury; ICU = Intensive Care Unit; SICU = Surgical Intensive Care Unit

More than half of the patients admitted to the SICU had a GCS score of < 9 and one-fourth of the patients had hypotension episodes at admission (Table 4).

**Table 4:** Vital signs characteristics at admission and ICU-mortality of patients in SICU, (N = 388)

Variables	Categories	Total (N = 388)	Outcome		
			Dead (n = 148)		Alive (n = 240)
Systolic blood pressure (mmHg)	< 90	100 (25.8%)		85 (57.4%)	15 (6.3%)
	90–140	267 (68.8%)		50 (33.8%)	217 (90.4%)
	> 140	21 (5.4%)		13 (8.8%)	8 (3.3%)
Heart rate (bpm)	< 60	17 (4.4%)		9 (6.1%)	8 (3.2%)
	60–100	100 (25.8%)		19 (12.8%)	81 (33.8%)
	> 100	271 (69.8%)		120 (81.1%)	151 (63%)
Respiratory rate (breath per minute)	< 12	6 (1.6%)		2 (1.4%)	4 (1.7%)
	12–20	30 (7.8%)		5 (3.4%)	25 (10.4%)
	> 20	352 (90.7%)		141 (95.2%)	211 (87.9%)
Temperature (^o^c)	< 36.5	92 (23.7%)		48 (32.4%)	44 (18.3%)
	36.5–37.5	124 (32.0%)		21 (14.2%)	103 (42.9%)
	> 37.5	172 (44.3%)		79 (53.4%)	93 (38.8%)
Saturation (SPO2) (%)	< 90	88 (22.7%)		66 (44.6%)	22 (9.2%)
	> 90	300 (77.3%)		82 (55.4%)	218 (90.8%)
Glasgow Coma Scale	Sever (3–8)	195 (50.3%)		135 (91.2%)	60 (25%)
	Moderate (9–13)	53 (13.6%)		7 (4.7%)	46 (19.2%)
	Mild (14–15)	140 (36.1%)		6 (4.1%)	134 (55.8%)

Key:- SICU = Surgical Intensive Care Unit; mmHg = millimeters of mercury; bpm = beats per minute; ^o^c= degrees centigrade

More than one–fourth of the participants had higher levels of potassium ( hyperkalemia) and sodium (hypernatremia) (27.1%), (28.8%) with an ICU-mortality of 83 (56.1%) and 43 (32.4%), respectively (Table 5).

**Table 5:** Investigations charactersitics and ICU-mortality of patients in SICU, (N = 388)

Variables	Categories	Total (N = 388)	Outcome	
			Dead (n = 148)	Alive (n = 240)
Hemoglobin (g/dl)	Anemia	321 (82.7%)	133 (89.9%)	188 (78.3%)
	Normal	67 (17.3%)	15 (10.1%)	52 (21.7%)
Random blood sugar (mg/dl)	< 70	41 (8.9%)	34 (23%)	7 (2.9%)
	70–160	139 (35.8%)	23 (15.5%)	116 (48.3%)
	> 160	208 (53.6%)	91 (61.5%)	117 (48.8%)
Creatinine (mg/dl)	< 0.7	39 (10%)	18 (12.2%)	21 (8.8%)
	0.7–1.4	224 (58.8%)	34 (23%)	190 (79.2%)
	≥ 1.5	125 (32.2%)	96 (64.8%)	29 (12%)
SGPT (U/L)	≤ 32	145 (37.4%)	27 (18.2%)	118 (49.2%)
	> 32	243 (62.6%)	121 (81.8%)	122 (50.8%)
SGOT (U/L)	≤ 32	96 (24.7%)	21 (14.2%)	75 (31.2%)
	> 32	292 (75.3%)	127 (85.8%)	165 (68.8%)
Sodium (mEq/L)	< 135	98 (25.3%)	62 (41.9%)	36 (15%)
	135–145	178 (45.9%)	38 (25.7%)	140 (58.3%)
	> 145	112 (28.8%)	48 (32.4%)	64 (26.7%)
Potassium (mEq/L)	< 3.5	49 (12.6%)	29 (19.6%)	20 (8.3%)
	3.5-5.0	234 (60.3%)	36 (24.3%)	198 (82.5%)
	> 5.0	105 (27.1) %	83 (56.1%)	22 (9.2%)

Key: - SICU = Surgical Intensive Care Unit; SGPT = Serum Glutamic-Pyruvic Transaminase; SGOT = Serum Glutamic-Oxaloacetic Transaminase; g/dl = grams per deciliter; mg/dl = milligrams per deciliter; mEq/L = milliEquivalents per Liter; U/L = Units per Liter

Among patients admitted to the unit, two hundred fifty-nine patients were supported by mechanical ventilation. Below 50% of the patients were on vasopressors and seven patients were treated with dialysis (Table 6).

**Table 6:** Interventions characteristics and ICU-mortality of patients in SICU, (N = 388)

Variables	Category	Total (N = 388)	Outcome	
			Dead (n = 148)	Alive (n = 240)
Mechanical ventilation	Yes	259 (66.8%)	145 (98%)	114 (47.5%)
	No	129 (33.2%)	3 (2%)	126 (52.5%)
Vasopressor	Yes	140 36.1%)	95 (64.2%)	45 (18.8%)
	No	248 (63.9%)	53 (35.8%)	195 (81.2%)
Transfusions	Yes	158 (40.7%)	85 (57.4%)	73 (30.4%)
	No	230 (59.3%)	63 (42.6%)	167 (69.6%)
Dialysis	Yes	7 (1.8%)	4 (2.7%)	3 (1.2%)
	No	381 (98.2%)	144 (97.3%)	237 (98.8%)
Thromboprophylaxis	Yes	163 (42%)	56 (37.8%)	107 (44.6%)
	No	225 (68%)	92 (62.2%)	133 (55.4%)
Feeding	Yes	140 (36.1%)	36 (24.3%)	104 (43.3%)
	No	248 (63.9) %	112 (75.7%)	136 (56.7%)
Fluid	Yes	380 (97.9%)	147 (99.3%)	233 (97.1%)
	No	8 (2.1%)	1 (0.7%)	7 (2.9%)
Reoperation	Yes	13 (3.6%)	5 (3.4%)	8 (3.3%)
	No	375 (96.4%)	143 (96.6%)	232 (96.7%)

Key: SICU = Surgical Intensive Care Unit

### Variables associated with ICU-mortality of patients admitted to surgical intensive care unit

The overall median follow-up time of patients at the intensive care unit was 264 (95% CI: 192,408) hours with a minimum and maximum follow-up time of 1 and 1152 h, respectively. In this study, 148 (38.1%) of the study participants died during the follow-up period in the surgical intensive care unit (SICU).

All variables entered into the bivariable Cox proportional hazard regression model. Trauma, the presence of complications, mechanical ventilation use, vasopressor support, blood transfusion, enteral feeding, reoperation, thromboprophylaxis, heart rate, oxygen saturation (SPO2), systolic blood pressure, temperature, SGPT, SGOT, creatinine, potassium level, sodium level, GCS, hemoglobin level, random blood sugar were fitted to multivariable Cox proportional hazard regression. Low GCS score, abnormal potassium level, hypoxia, mechanical ventilation, absence of enteral feeding, and absence of thromboprophylaxis were variables associated with ICU-mortality among patients admitted to the SICU (Table 7).

**Table 7:** Bivariable and multivariable Cox regression analysis of variables associated with ICU-mortality among patients in the SICU (N = 388)

Variables	Categories	Outcome		CHR (95%CI)	AHR (95%CI)	p-value
		Dead (n = 148 )	Alive (n = 240)			
Glasgow coma scale	Sever	135	60	14.43 (6.36–32.73)*	4.06(1.51–10.89)	0.005
	Moderate	7	46	2.50 (0.84-7.47)*	0.97(0.30–3.15)	
	Mild	6	134	1	1	
Mechanical ventilation	Yes	145	114	4 (1–15)*	12 (3–45)	0.002
	No	3	126	1	1	
Potassium	< 3.5	29	20	3.98 (2.43–6.51)*	3.46(1.83–6.55)	< 0.001
	3.5-5.0	36	198	1	1	
	> 5.0	83	22	6.50 (4.39–9.63)*	2.41(1.29–4.51)	0.005
Saturation (Spo_2_)	< 90	66	22	4.26 (3.06–5.94)*	1.66(1.10–2.48)	0.014
	>=90	82	218	1	1	
Feeding	Yes	36	104	1	1	
	No	112	136	4.29(2.88–6.39)*	3.56(2.20–5.78)	< 0.001
Thromboprophylaxis	Yes	56	107	1	1	
	No	92	133	7.07(4.62–10.81)*	10.8(6.0419.29)	< 0.001

Key: - * Significant (P-value < 0.05); CHR = Crude Hazard Ratio; AHR = Adjusted Hazard Ratio; 1 = reference categories, SICU = Surgical Intensive Care Unit

## Discussion

This study was conducted to identify variables associated with ICU-mortality among patients admitted to the surgical intensive care unit at a Comprehensive Specialized Hospital in Ethiopia.

In this study, emergency cases and males had a higher admission rate to the surgical intensive care unit. This study is inline in studies conducted in Ireland, Jordan, and India [24–26].

This could be explained by the higher incidence of trauma (violence) in males and the insufficient time for optimisation in emergency patients.

Trauma was the leading cause of admission to surgical intensive care unit (SICU) which was in line with studies performed in Western Kenya, Aqaba-Jordan, Tribhuvan University, Nepal, and Ethiopia [24, 27–29], respectively. However, other studies in Ethiopia, Nigeria, Malawi, Ireland, and United States [14, 21, 26, 30, 31] showed that the leading cause of admission to SICU was complications after acute abdomen and thoracoabdominal injuries. Early warning scores have been established as clinical prognostication tools to identify patients who are fast deteriorating [32].

The ICU-mortality of patients admitted to the surgical intensive care unit was 38.1% (95% CI: 33.3–42.1). This finding is consistent with the findings of other studies conducted in Nigeria (34.6%) [10], Uganda (37.6%) [33], Tanzania (41.4%) [34], the Republic of Congo (37.4%) [35], St. Paul’s Hospital Millennium Medical College (40.2%)[14], and Jimma (39.8%) [19]. This similarity can be explained by the fact that the places have comparable resources and population demographics.

However, this study showed a higher frequency of ICU-mortality of patients in SICU than studies conducted in Mekele (Ethiopia) (27%) [36], three Scandinavian countries (9.1%) [37], Spain (5.63%) [38], United States (11.2%) [39], Turkey (15.8%) [40], Brazil (22.9%) [41], and Greece (27.3%) [42]. The reason might be due to a lack of necessary equipment (like arterial blood gas (ABG)-analyzer machine, portable dialysis machine, and portable x-ray service), pre-hospital care, or a general lack of resources in the area of the current study.

This result, however, is lower than studies carried out in Turkey (50%) [43], Egypt (58%) [44], Jimma (52.8%) [45], and Brazil (89.1%) [46]. This study also lower compared to another study conducted in Western Kenya showed that 30-day mortality was 57.3% [11]. This can be explained that their report was 30-day mortality.

In our study, the median length of ICU stay was 264 h (11 days) and in line with studies conducted at St. Paul’s Hospital Millennium Medical College [14], Belgium, Nigeria, Tanzania, Malawi, and Kenya [5, 21, 30, 47, 48], respectively. However, it is much longer than studies performed in Kenya and Tanzania [29, 47], respectively. The reason could be due to the scarcity of necessary resources in a setting like a high-dependency unit.

The hazard of death among patients with a GCS score < 9 was higher as compared to those who had a higher GCS score. This finding is in agreement with other studies conducted in Boston, Greece, Turkey [40, 43], Spain [49], Egypt, and Ethiopia.

Patients who were on mechanical ventilation had a higher risk of ICU-mortality. This finding is in line with studies done at Saint Paul Hospital Millennium Medical College (Ethiopia), Kenya, and Brazil [29, 50, 51], respectively. The possible reason might be related to an increased severity of these patients. To be added, mecanichal ventilation leads to increased risk of ventilator-associated pneumonia and other nosocomial infection [52, 53].

The risk of death among patients with hypoxia had higher as compared with patients without hypoxia, in line with previous studies [54–56]. As a result, patients may benefit from prompt management of hypoxia by the use of oxygen with higher F_i_O_2_ up to 100% oxygen or assist with mechanical ventilation accordingly [57].

This study also found that hypokalemia was another variable associated with ICU-mortality among patients admitted to SICU. This study is in line with the studies conducted in Japan, China [58], and Boston[59]. Hyperkalemia was also another variable associated with ICU-mortality of patients in SICU, in line with other similar studies conducted in the Netherlands, Boston, US [59–61], respectively. The evidence suggested that potassium is an important determinant of myocardial function and hypokalemia can lead to arrhythmia and sudden cardiac death [62, 63].

The hazards of death among patients who didn’t get thromboprophylaxis had 10.53 times higher as compared with patients getting thromboprophylaxis as already highlighted by previous studies [64, 65]. Due to their immobility, use of mechanical ventilation, and central catheters, patients in the intensive care unit (ICU) had a higher risk of thrombotic events. However, the requirement for thromboprophylaxis decreased the dangers of death in the ICU [66].

Interestingly, enteral nutrition seems to represent a preventive factor of ICU-mortality among patients admitted to the SICU, as the risk of death among patients receiving enteral nutrition was decreased by 77% compared with those who didn’t receive enteral nutrition. In fact, it is known that enteral nutrition provides both macro and micronutrients, which help to sustain gut integrity through the stimulation of blood flow in intraepithelial cells, and promote immune functions which decreases the risk of infection [67, 68].

### Limitations of the study

Using secondary data may limit the researcher to measure all possible predictors like body mass index (BMI), pain management, and sedation were not assessed because of a lack of organized documentation. Physiologic scores such as acute physiological and chronic health evaluation (APACHE) and sequential organ failure assessment (SOFA) were not obtained. Additionally, the small sample size, collected in a single-center, as well as the lack of generalisability, and changes in practice over time must be highlighted as other relevant limations of the study.

Finally, we used as primary outcome ICU-mortality, as we were unable to provide 28-days mortality.

## Conclusions

The overall ICU-mortality of patients admitted to surgical intensive care unit at academic hospital in Ethiopia was higher compared to patients admitted in similar intensive care unit type in developed countries. Septic shock was the leading cause of death. Hypokalemia, hyperkalemia, lower GCS score(< 9), mechanical ventilation, hypoxia, absence of thromboprophylaxis, and absence of enteral feeding were variables associated with ICU-mortality among patients admitted to surgical intensive care unit.

### Electronic supplementary material

Below is the link to the electronic supplementary material.

Supplementary Material 1

## Data Availability

The data sets used and analyzed during the study are available from the corresponding author/primary investigator upon reasonable request.
