# Supplementary material for: Variables associated to intensive care unit (ICU)-mortality among patients admitted to surgical intensive care unit in Ethiopia: a retrospective observational study
Source: BMC Anesthesiol. 2023 Aug 18;23:279. doi: 10.1186/s12871-023-02230-w (PMC10436438; doi:10.1186/s12871-023-02230-w)
Supplement: Supplementary file 1 — Supplementary Material 1 [file 12871_2023_2230_MOESM1_ESM.docx]

**Operational Definitions**

**Censored:** Those patients who were admitted to the ICU but who were released against physicians' orders/signed not to get ICU care for a number of reasons, including a lack of insurance or a poor prognosis for the condition.

**Incomplete records:** the presence of missing necessary information in patients, such as missing the outcome variable or the date of the outcome variable.

**Event:** the occurrence of death among surgical patients from admission to discharge.

**Survival time:** Measures the follow-up time from a defined starting point/from the admission of a surgical patient admitted to ICU up to the event’s occurrence.

**Follow-up time:** from the time of ICU admission to the occurrence of the event or censorship.

**Length of ICU stay (LOS):** a period in hours that patients stayed in ICU from admission to discharge.

**Hypokalemia:** the level of potassium <3.5mmol/l and also Hyperkalemia: the level of potassium >5.0mmol/l.

**Clinical outcome:** indicated either patients survived or died at the time of discharge**.**

**ICU Mortality:** is calculated as the number of deaths of patients given particular diagnoses divided by the total number of patients with that diagnosis.

**Hypoxia:** is defined as oxygen saturation *<*90% at any time point during ICU admission.

**Anemia** is a condition in which the number of red blood cells or the hemoglobin concentration within them is lower than normal (anemia in adults is a hemoglobin (Hgb) value of less than 12 g/dl).

**The Glasgow Coma Scale (GCS**)- classifies Traumatic Brain Injuries (TBI) as Mild (14–15); Moderate (9–13) or Severe (3–8).
